# Diurnal Variation of Real-Life Insulin Sensitivity Factor Among Children and Adolescents With Type 1 Diabetes Using Ultra-Long-Acting Basal Insulin Analogs

**DOI:** 10.3389/fped.2022.854972

**Published:** 2022-03-08

**Authors:** Ahmed M. Hegab

**Affiliations:** Department of Pediatrics, Faculty of Medicine, Sohag University, Sohag, Egypt

**Keywords:** children and adolescents, insulin sensitivity factor, multiple daily injection, type 1 diabetes, insulin analogs

## Abstract

**Background:**

Estimation of insulin sensitivity factor (ISF) is essential for correction insulin doses calculation. This study aimed to assess real-life ISF among children and adolescents with type 1 diabetes using ultra-long-acting basal insulin analogs and to detect factors associated with ISF among those patients.

**Methods:**

This prospective observational study was conducted at Sohag University Hospital, Egypt, and included 93 participants aged 6–18 years, diagnosed with T1DM for at least 1 year and using insulin glargine 300 Units/mL or insulin degludec 100 Units/mL as basal insulin. The ISF, insulin-to-carbohydrate ratio (ICR) and insulin doses were initially assessed then adjusted as required. The participants were regularly contacted throughout the follow-up period. Glycemic control parameters were assessed after 3 months.

**Results:**

The ISF showed diurnal variation with higher correction dose requirements for the morning than for the rest of the day (*p* < 0.001). This pattern of diurnal variation was found in participants with different pubertal stages and in participants using either type of ultra-long acting basal insulin analogs. There was no significant difference between the ISF calculated according to the 1800 rule [1800/Total daily insulin dose (TDD)] and the morning ISF (*p* = 0.25). The 1800 rule-calculated ISF was significantly lower than the actual ISF for the afternoon (*p* < 0.001) and the evening (*p* < 0.001). ISF at different times of the day were significantly correlated with age, body mass index, pubertal stage, diabetes duration, TDD, and ICR. Multiple regression analysis revealed that ICR was the most significant factor associated with ISF. Linear regression analysis revealed that the ISF (in mg/dL) for any time of the day could be estimated as 5.14 × ICR for the same time of the day (coefficient = 5.14, 95% confidence interval: 5.10–5.19, *R*^2^ = 0.95, *p* < 0.001).

**Conclusion:**

Diurnal variation of ISF that had to be considered for proper calculation of correction doses. This diurnal variation was found in children and adolescents with different pubertal stages. The 1800 rule was appropriate for the morning correction doses but not in the afternoon or the evening. The TDD and the ICR could be used for ISF estimation.

## Introduction

Appropriate estimations of insulin sensitivity factor (ISF) and insulin-to-carbohydrate ratio (ICR) in children and adolescents with type 1 diabetes mellitus (T1DM) using intensive insulin therapy are essential for proper calculation of bolus insulin doses (1). In patients using either multiple daily injection (MDI) therapy or insulin pump therapy, bolus insulin doses are given as pre-meal doses that cover the carbohydrate content in the meals and as correction doses given to reduce high blood glucose levels down to the target range (2).

The pre-meal dose is calculated as the amount of carbohydrate in grams of a certain meal divided by the ICR for that meal. ICR is defined as the grams of carbohydrates that can be covered by one unit of insulin (3). Calculation of ICR is essential to give the patients the flexibility of eating different meal sizes and adjusting the pre-meal bolus doses according to the amount of carbohydrate in each meal (2). Moreover, correction doses are calculated as the difference between the current blood glucose level and the target blood glucose level divided by the ISF which is defined as the amount of reduction of blood glucose levels (in mg/dL or mmol/L) that can be achieved by 1 unit of insulin (3, 4). Estimation of ISF allows proper determination of correction doses required to prevent prolonged periods of hyperglycemia that impair the glycemic control and result in elevated glycosylated hemoglobin A1c (HbA1c) levels (2).

Some studies were conducted to assess the ICR in children and adolescents with T1DM and proposed different formulas for ICR estimation in patients using either insulin pumps (5–9) or MDI regimen (10). However, only a few studies were done to assess the ISF in pediatric patients with T1DM. The 1500 rule and the 1800 rule are often used for the initial estimation of ISF (1, 2). Davidson et al., proposed the 1500 rule based on their clinical experience on adult patients using short-acting insulin (11, 12). According to this rule, the ISF (in mg/dL) is calculated as 1500 divided by the total daily insulin doses (TDD) which is the sum of basal and bolus insulin doses used by the patient throughout the day (1, 2). For estimation of ISF in mmol/L, the 83 rule (83/TDD) has been used (1). With the introduction of the rapid-acting insulin analogs, the 1800 rule and the 100 rule were proposed for calculation of ISF in mg/dL and mmol/L, respectively (9).

Although the 1800 rule (the 100 rule in mmol/L) is frequently used for ISF estimation, some studies conducted on children and adolescents using insulin pump therapy reported that the actual correction doses needed for those patients were significantly lower than those calculated by the 1800 rule (7, 9). Moreover, Hanas et al., reported that ISF in prepubertal children using insulin pumps was lower with higher bolus insulin requirements in the morning than for the rest of the day (9). This circadian change in ISF has to be reflected in the proposed formula for ISF estimation.

With the introduction of ultra-long-acting basal insulin analogs, the currently used formulas for ISF estimation need to be re-evaluated. Insulin glargine 300 Units/mL and insulin degludec 100 Units/mL have improved action profiles that are similar to the physiologic endogenous basal insulin secretion (13) with prolonged durations of action lasting for more than 24 h and even distribution of insulin activity throughout the day with less day-to-day variability and within-day fluctuations in insulin activity (14). The different pharmacokinetic and pharmacodynamic profiles of these ultra-long-acting basal insulin analogs might affect the TDD and consequently the ISF.

Furthermore, insulin sensitivity in children and adolescents with T1DM might be affected by several factors. Poor glycemic control was associated with decreased insulin sensitivity (15). Moreover, insulin sensitivity decreased with age and with the onset of puberty (6, 16). Several studies reported that pubertal and postpubertal adolescents have more insulin resistance than prepubertal children (17–19). Some studies reported that there were differences in insulin resistance between girls and boys during puberty (20, 21). However, the sex differences in insulin sensitivity might be attributed to differences in adipose tissue distribution (22, 23). Furthermore, increased body mass index (BMI) was correlated with decreased insulin sensitivity (24). The decrease in insulin sensitivity was related more to abdominal than peripheral obesity (25, 26). Increased waist circumference which could be used as a clinical marker for abdominal obesity (27) was associated with increased insulin resistance in children (28) and adolescents (29). In addition, changes in insulin sensitivity occur throughout the day with decreased insulin sensitivity in the early morning hours which is known as the dawn phenomenon (1). This diurnal change in insulin sensitivity might be related to the time and the magnitude of different hormones secretions, especially growth hormone and glucocorticoids. These hormones might decrease insulin sensitivity by increasing hepatic glucose production and decreasing glucose uptake at peripheral tissue (1, 30, 31).

Therefore, this study aimed to assess the ISF at different times of the day among children and adolescents with T1DM using ultra-long-acting basal insulin analogs. The study also aimed to detect factors associated with ISF and to find out appropriate methods for ISF estimation among those patients.

## Materials and Methods

### Study Settings

This was a prospective observational study conducted over 1 year (from September 2020 to August 2021) at the pediatric diabetes clinic, Sohag University Hospital, Sohag, Egypt.

The pediatric diabetes unit at Sohag University Hospital is the only specialized pediatric diabetes unit in Sohag Governorate in Upper Egypt which has a population of about 5.5 million. There are about 350 to 400 children and adolescents with T1DM at regular follow-up at the pediatric diabetes clinic annually. The clinic is held twice weekly and about 25 to 30 children and adolescents with T1DM are seen at the clinic every week. At the time of the study, there were two pediatric endocrinology consultants, a specialized dietitian and three rotating pediatric specialist registrars working at the unit.

The majority of patients followed up at the clinic are using the MDI regimen and finger-stick glucometers. The percentages of patients using insulin pumps or continuous glucose monitoring (CGM) devices are very small because the national health insurance system in Egypt still does not cover the costs of insulin pumps and CGM devices. Only 11 patients using insulin pumps and 18 patients using CGM devices were followed up at the clinic at the time of the study.

### Study Population

Children and adolescents aged 6–18 years, diagnosed with T1DM for more than 1 year, using the MDI regimen with insulin glargine 300 Units/mL or insulin degludec 100 Units/mL as basal insulin and using advanced carbohydrate counting methods for at least 6 months, were included in the study. Children and adolescents with associated celiac disease or autoimmune hypothyroidism were excluded.

The participants were consecutively recruited from the pediatric diabetes clinic. Among 357 children and adolescents with T1DM who were at regular follow-up at the pediatric diabetes clinic throughout the study period, 342 patients were assessed for eligibility. One hundred eighty-three patients fulfilled the inclusion criteria. Thirty patients were excluded (17 patients had associated autoimmune hypothyroidism and 13 patients had associated celiac disease). Among the 153 patients eligible for participation, the parents/legal guardians of 102 patients accepted to participate in the study. Nine participants lost follow-up after participation and were excluded from the analysis. The remaining 93 participants completed the follow-up period and were included in the analysis. None of the study participants used CGM devices.

### Ethical Considerations

The study protocol was approved by the Research Ethics Committee at Sohag Faculty of Medicine. Written informed consents were obtained from the parents or legal guardians of all study participants.

### Assessment of the Participants

The study participants were subjected to full history taking and thorough clinical examination at the pediatric diabetes clinic and their medical files were reviewed.

The BMI standard deviation scores (SDS) were calculated using the World Health Organization (WHO) standard references (32). The pubertal status was assessed and staged according to the Tanner stages (33, 34). The study participants were classified according to their Tanner stages into 3 groups; prepubertal participants (Tanner stage 1), early to mid-pubertal participants (Tanner stages 2 and 3) and late-pubertal and postpubertal participants (Tanner stages 4 and 5).

### Management of the Participants

The study participants received the standard management for T1DM used in our pediatric diabetes unit. The management included the use of carbohydrate counting methods, intensive insulin therapy and frequent blood glucose monitoring.

### Carbohydrate Counting

In our pediatric diabetes unit, children and adolescents with T1DM and their families are educated about carbohydrate counting and different methods to calculate the amount of carbohydrate in each meal or snack at the diagnosis of T1DM. The carbohydrate counting skills of the patients and their families are checked regularly at each follow-up visit by the dietitian. The daily caloric requirements and the recommended daily amount of carbohydrates consumed by children and adolescents with T1DM are determined by the dietitian according to the patients’ age, sex and activity levels (35).

The study participants used either the gram increments method or the 15-gram carbohydrate exchange method for carbohydrate counting (36). They were advised to keep their daily caloric intake within the recommended ranges (35) and to have approximately 50% of their daily calories as carbohydrates (36).

### Intensive Insulin Therapy

The study participants used rapid-acting insulin analogs [insulin lispro (Humalog), insulin aspart (Novorapid) or insulin glulisine (Apidra)] as bolus insulin for pre-meal doses and correction doses. Bolus insulin was given by either one unit or half-unit increments pens according to the participant insulin requirements. The study participants used ultra-long-acting insulin analog [insulin degludec 100 Units/mL (Tresiba) or insulin glargine 300 Units/mL (Toujeo)] as once-daily basal dose at night (8–10 PM). The participants used 4–6 mm needles for insulin injections. They were instructed to rotate injections regularly among different sites and to avoid injections in lumpy areas.

The pre-meal bolus dose was calculated as the amount of carbohydrate in grams in the meal divided by the ICR for that meal. ICR was assessed for each meal and it was considered accurate if the blood glucose level 2 h after the meal remained within 30 mg/dL of the pre-meal level (1, 10).

The correction doses were calculated as (the current blood glucose level in mg/dL −120 mg/dL)/ISF, aiming at achieving blood glucose levels between 90 and 150 mg/dL 2 h after the correction dose. The correction doses were given either in association with the pre-meal doses or separately if the blood glucose levels were above the target range. However, at least a 2–3-h interval was allowed between each two consecutive bolus doses to avoid insulin stacking induced by repeated injections at short intervals (9, 37). The ISF (in mg/dL) was initially estimated by the 1800 rule then adjusted according to the blood glucose measurements. The ISF was considered accurate if the blood glucose level returned to the target range 2 h after the correction dose (1).

The ISF for the study participants was assessed for different times of the day separately. The morning ISF was used for correction doses given between 6 AM and 12 PM. The afternoon ISF was used for correction doses given between 12 PM and 6 PM. The evening and nighttime ISF was used for correction doses given after 6 PM.

### Blood Glucose Monitoring

In our pediatric diabetes unit, children and adolescents with T1DM are instructed to measure their blood glucose levels at home by finger-stick glucometer before and 2 h after each meal, at bedtime and at 3 AM and to record their glucometer measurements, carbohydrate grams or portions consumed in each meal or snack, the pre-meal bolus doses and correction doses in their logbooks. The target blood glucose ranges are set according to the International Society for Pediatric and Adolescent Diabetes (ISPAD) clinical practice guideline recommendations (38). The pre-meal target is 70–130 mg/dL, 2 h postprandial target is 90–180 mg/dL, bedtime target is 80–140 mg/dL and overnight target is 80–162 mg/dL. Adjustments of basal insulin doses are done to keep fasting blood glucose levels in the morning between 70 and 130 mg/dL without nocturnal hypoglycemia.

Moreover, children and adolescents with T1DM and their families are given written information about the symptoms of hypoglycemia and how to deal with it at home based on the ISPAD clinical practice guideline recommendations (39). They are also given written instructions about blood glucose and urinary ketone monitoring as well as insulin doses and carbohydrate intake adjustments before, during and after physical exercises (40) and during intercurrent illnesses (41) according to the ISPAD clinical practice guidelines recommendations.

The study participants and their families were instructed to keep recording their insulin doses, carbohydrate contents of different meals or snacks and blood glucose measurements regularly in their logbooks. They were also asked to document any attack of severe hypoglycemia. Severe hypoglycemia was defined as an episode of a blood glucose level below 70 mg/dL associated with severe cognitive impairment with the need for external assistance by another person to correct hypoglycemia (39).

### Follow Up of the Study Participants

The study participants and their families were contacted at the end of the first week of their participation in the study either by direct telephone calls or through the WhatsApp smartphone communication application (WhatsApp Inc., Mountain View, CA, United States) to check that the basal insulin doses, ICR and ISF used by them were accurate and adjustments were done if required. The participants were contacted every month for the next 3 months and their logbook data were reviewed. The insulin doses were adjusted if needed. The study participants were encouraged to contact the pediatric diabetes management team at Sohag University Hospital whenever they had any difficulties concerning the control of their diabetes.

### Outcome Measures

At the end of the 3 months follow-up period, the participants were reviewed at the pediatric diabetes clinic. The data recorded in their logbooks were checked. The actual ISF, ICR and basal insulin doses used by the study participants at the third-month follow-up visit were used for the analysis. For variables that varied from day to day as the TDD and the daily correction dose, the average of the doses recorded in the last week before the follow-up visit approximated to the nearest unit was used for the analysis.

Hemoglobin A1c levels were measured at the follow-up visit using high-performance liquid chromatography (Bio-Rad D-10; Bio-Rad Laboratories, Hercules, CA, United States) calibrated against the National Glycosylated Standardization Program (NGSP). The study participants were considered to have optimum glycemic control if their HbA1c levels were below 7.5% and they had no attacks of severe hypoglycemia throughout the follow-up period.

### Statistical Analysis

Statistical analysis was performed using IBM SPSS Statistics for Windows, version 22.0 (IBM Corp., Armonk, NY, United States). The Kolmogorov−Smirnov test was used to assess the normality of distribution for continuous variables. Normally distributed continuous variables were expressed as means ± standard deviations (SD). Continuous variables with non-parametric distributions were presented as medians [interquartile ranges (IQR)]. Categorical variables were expressed as numbers and percentages.

For comparisons between two groups of continuous variables, the independent sample *t*-test was used for variables with normal distribution, and the Mann-Whitney U test was used for variables with non-parametric distributions. The Chi-square test was used to compare two groups of categorical variables. Wilcoxon signed-rank test was used to compare the 1800 rule-calculated ISF with the actual ISF detected in the study participants. It was also used to compare the ISF as well as the ICR between different times of the day. For comparisons between prepubertal, early to mid-pubertal and postpubertal participants, the one-way analysis of variance (ANOVA) was used for normally distributed variables and the Kruskal-Wallis test was used for non-normally distributed variables. Spearman’s rank correlation coefficient was used to measure the degree of association between ISF for different times of the day and the age, sex, Tanner stage, BMI, duration of diabetes, TDD, ICR, and the HbA1c levels for the study participants.

To identify factors associated with ISF, factors that were significantly correlated with ISF at different times of the day were included in multiple regression analysis models. Initially, bivariate correlations were used to assess the association between each two of these variables. If Spearman’s correlation coefficient between two variables was above 0.9, one of these two variables was excluded from further analysis. The TDD and Tanner stage were excluded on this basis in favor of the ICR and the age, respectively. Univariate analysis was performed to identify the association between each of the included variables and ISF. Variables that showed significant association in univariate analysis were entered into a multivariate analysis model to assess the simultaneous effects of these variables. Linear regression analyses between ISF and each of the TDD and ICR were also used to derive formulas for ISF estimation. *P*-value < 0.05 was considered statistically significant.

## Results

Ninety-three participants were included in the study. The clinical characteristics of the study participants are shown in Table 1. There were no significant differences in age, sex, BMI SDS, duration of diabetes, pubertal stages or the type of the rapid-acting insulin between participants using insulin degludec 100 Units/mL and those using insulin glargine 300 Units/mL.

**TABLE 1:** Baseline clinical characteristics of the study participants.

	All participants (*n* = 93)	Participants using insulin degludec 100 Units/mL (*n* = 49)	Participants using insulin glargine 300 Units/mL (*n* = 44)	*p*-Value
Age (years), median (IQR)	10.5 (8.0–13.0)	11.0 (8.5–12.8)	10.5 (7.5–14)	0.76
Sex Male, *n* (%) Female, *n* (%)	46 (49.5%) 47 (50.5%)	25 (51.0%) 24 (49.0%)	21 (47.7%) 23 (52.3%)	0.75
Duration of diabetes (years), median (IQR)	3.0 (2.0–5.0)	3.5 (2.0–5.0)	3.0 (2.4–5.4)	0.89
BMI SDS, median ± SD	0.55 ± 0.83	0.60 ± 0.87	0.51 ± 0.79	0.61
Pubertal stage; Tanner stage 1, *n* (%) Tanner stage 2, *n* (%) Tanner stage 3, *n* (%) Tanner stage 4, *n* (%) Tanner stage 5, *n* (%)	40 (43.0%) 13 (14.0%) 16 (17.2%) 15 (16.1%) 9 (9.7%)	20 (40.8%) 8 (16.3%) 8 (16.3%) 10 (20.4%) 3 (6.1%)	20 (45.5%) 5 (11.4%) 8 (18.2%) 5 (11.4%) 6 (13.6%)	0.54
Type of bolus insulin used: Insulin glulisine, *n* (%) Insulin lispro, *n* (%) Insulin aspart, *n* (%)	29 (31.2%) 32 (34.4%) 32 (34.4%)	16 (32.7%) 18 (36.7%) 15 (30.6%)	13 (29.5%) 14 (31.8%) 17 (38.6%)	0.71

*BMI, body mass index; IQR, interquartile range; SD, standard deviation; SDS, standard deviation score.*

Table 2 shows insulin doses, ICR and glycemic control parameters for the study participants. There were no statistically significant differences between participants using insulin degludec 100 Units/mL and those using insulin glargine 300 Units/mL as regards TDD, basal insulin doses, correction doses, ICR, HbA1c levels or the frequency of severe hypoglycemia. The ICR for the study participants showed diurnal variation with lower ICR and consequently higher premeal bolus dose requirements in the morning compared to the afternoon (*p* < 0.001) and the evening (*p* < 0.001). The evening ICR was significantly higher compared to the afternoon ICR (*p* < 0.001). This pattern of diurnal variation of ICR was found in participants using insulin degludec 100 Units/mL and those using insulin glargine 300 Units/mL.

**TABLE 2:** Insulin doses, insulin-to-carbohydrate ratio, and glycemic control parameters for the study participants.

	All participants (*n* = 93)	Participants using insulin degludec 100 Units/mL (*n* = 49)	Participants using insulin glargine 300 Units/mL (*n* = 44)	*p*-value
TDD (U/kg/day), median (IQR)	0.93 (0.87–0.98)	0.94 (0.86–1.00)	0.93 (0.88–0.97)	0.53
Basal insulin dose (U/kg/day), median (IQR)	0.41 (0.36–0.45)	0.41 (0.36–0.46)	0.41 (0.36–0.44)	0.31
Basal dose to TDD (%), mean ± SD	44.1 ± 3.6	44.4 ± 3.1	43.7 ± 4.1	0.37
Correction dose to TDD (%), mean ± SD	8.2 ± 2.3	7.9 ± 2.2	8.4 ± 2.3	0.28
Morning ICR (grams/unit of insulin), median (IQR)	10 (7.5–15)	10 (7–12)	12 (7.5–15)	0.42
Afternoon ICR (grams/unit of insulin), median (IQR)	12 (7.5–15)	12 (7.5–15)	12 (7.5–15)	0.33
Evening ICR (grams/unit of insulin), median (IQR)	15 (7.5–15)	15 (7.5–15)	15 (10–15)	0.57
HbA1c (%), median (IQR)	8.4 (7.3–9.3)	8.3 (7.2–9.2)	8.4 (7.4–9.3)	0.53
Participants with attacks of severe hypoglycemia, *n* (%)	12 (12.9%)	5 (10.2%)	7 (15.9%)	0.41
Participants with optimum glycemic control, *n* (%)	29 (31.2%)	16 (32.7%)	13 (29.5%)	0.74

*HbA1c, glycosylated hemoglobin A1c; ICR, insulin-to-carbohydrate ratio; IQR, interquartile range; SD, standard deviation; TDD, total daily insulin dose.*

The ISF in mg/dL and the insulin sensitivity constants (calculated as TDD in units/day × ISF in mg/dL) for the study participants are shown in Table 3. There were no significant differences in ISF or insulin sensitivity constants between participants using insulin degludec 100 Units/mL and those using insulin glargine 300 Units/mL at different times of the day. ISF showed diurnal variation with significantly lower ISF and consequently higher correction dose requirements in the morning compared to the afternoon (*p* < 0.001) and the evening (*p* < 0.001). Moreover, the evening ISF was significantly higher compared to the afternoon ISF (*p* < 0.001). This pattern of diurnal variation of ISF was found in participants using either type of ultra-long-acting basal insulin analogs. There was no significant difference between ISF calculated according to the 1800 rule and the real-life morning ISF (*p* = 0.25). However, the 1800 rule-calculated ISF were significantly lower compared to the actual ISF in the afternoon (*p* < 0.001) and the evening (*p* < 0.001).

**TABLE 3:** The insulin sensitivity factors (ISF) and the insulin sensitivity constants (calculated as the ISF in mg/dL × total daily insulin dose in units/day) for the study participants.

	All participants (*n* = 93)	Participants using insulin degludec 100 Units/mL (*n* = 49)	Participants using insulin glargine 300 Units/mL (*n* = 44)	*p*-value
The “1800” rule-calculated ISF (in mg/dL), median (IQR)	56.3 (36-72)	56.2 (35.3-64.3)	58.1 (36.3-77.4)	0.45
Real-life morning ISF (in mg/dL), median (IQR)	50 (40-75)	50 (40-60)	60 (40-75)	0.53
Real-life afternoon ISF (in mg/dL), median (IQR)	60 (40-75)	60 (40-75)	60 (40-78.8)	0.38
Real-life evening ISF (in mg/dL), median (IQR)	75 (45-77.5)	75 (40-75)	75 (50-80)	0.47
Morning insulin sensitivity constant, median (IQR)	1800 (1680-1920)	1800 (1680-1922.5)	1780 (1620-1915)	0.47
Afternoon insulin sensitivity constant, median (IQR)	1920 (1800-2075)	1920 (1800-2032)	1910 (1760-2100)	0.98
Evening insulin sensitivity constant, median (IQR)	2100 (1920-2250)	2100 (1965-2275)	2100 (1905-2250)	0.55

*ISF, insulin sensitivity factor; IQR, interquartile range.*

Table 4 shows the insulin doses, ICR and ISF for the study participants according to their pubertal status. Late-pubertal and postpubertal participants had significantly higher TDD compared to prepubertal and early to mid-pubertal participants (*p* = 0.004). However, there were no significant differences between prepubertal, early to mid-pubertal and late-pubertal and postpubertal participants as regards the daily basal insulin dose or the daily correction dose to TDD ratio (*p* = 0.69 and 0.26, respectively). The ISF and ICR for different times of the day were significantly lower in late-pubertal and postpubertal participants reflecting higher bolus insulin requirements in this group of participants compared to the prepubertal and the early to mid-pubertal participants (*p* < 0.001).

**TABLE 4:** Insulin doses, insulin-to-carbohydrate ratio and insulin sensitivity factor for the study participants according to their pubertal status.

	Prepubertal (Tanner stage 1) (*n* = 40)	Early to mid-pubertal (Tanner stages 2 and 3) (*n* = 29)	Late-pubertal and postpubertal (Tanner stages 4 and 5) (*n* = 24)	*p*-value
TDD (U/kg/day), median (IQR)	0.91 (0.85 – 0.95)	0.94 (0.89 – 1.01)	0.97 (0.91 – 1.04)	0.004
Basal insulin dose (U/kg/day), median (IQR)	0.40 (0.35–0.44)	0.41 (0.38–0.45)	0.42 (0.37–0.47)	0.69
Correction dose to TDD (%), mean ± SD	8.3 ± 2.5	8.6 ± 2.2	7.6 ± 1.7	0.26
Morning ICR (grams/unit of insulin), median (IQR)	15 (12–15)	10 (7.5–12)	6.25 (5.00–7.50)	< 0.001
Afternoon ICR (grams/unit of insulin), median (IQR)	15 (12–15)	10 (7.5–12)	6.25 (6.25–7.50)	< 0.001
Evening ICR (grams/unit of insulin), median (IQR)	15 (15–18)	12 (10–15)	7.5 (6.4–9.4)	< 0.001
Real-life morning ISF (in mg/dL), median (IQR)	75 (60–80)	50 (40–60)	35 (30–40)	< 0.001
Real-life afternoon ISF (in mg/dL), median (IQR)	75 (63.8–80.0)	50 (40–60)	40 (30–40)	< 0.001
Real-life evening ISF (in mg/dL), median (IQR)	80 (75–90)	60 (50–75)	40 (40–50)	< 0.001

*ICR, insulin-to-carbohydrate ratio; IQR, interquartile range; ISF, insulin sensitivity factor; SD, standard deviation; TDD, total daily insulin dose.*

Diurnal variations of ICR and ISF were found in participants with different pubertal stages. The ICR and ISF were significantly higher in the morning compared to the afternoon in prepubertal (*p* = 0.003 and 0.002, respectively), early to mid-pubertal (*p* = 0.005 and 0.003, respectively) and late-pubertal and postpubertal participants (*p* = 0.011 and 0.01, respectively). Similarly, the ICR and ISF were significantly higher in the morning compared to the evening in prepubertal (*p* < 0.001), early to mid-pubertal (*p* < 0.001) and late-pubertal and postpubertal participants (*p* < 0.001). Moreover, the ICR and ISF were significantly lower in the evening compared to the afternoon in prepubertal (*p* < 0.001), early to mid-pubertal (*p* = 0.001 and 0.004, respectively) and late-pubertal and postpubertal participants (*p* < 0.001 and 0.002, respectively).

Table 5 shows the correlations between ISF for different times of the day and the age, sex, Tanner stage, BMI, duration of diabetes, TDD, ICR, and the HbA1c levels for the study participants. There were strong negative correlations between ISF for different times of the day and the age, Tanner stage, BMI, duration of diabetes and TDD of the study participants and a strong positive correlation between ISF for different times of the day and the ICR for the same time of the day. There were no significant correlations between ISF at different times of the day and the sex or the HbA1c levels. Multiple linear regression analyses of factors associated with ISF at different times of the day are shown in Table 6. The most significant factor associated with ISF was the ICR for the same time of the day.

**TABLE 5:** Correlations between the insulin sensitivity factors for different times of the day and some clinical variables of the study participants.

Variables	Morning ISF		Afternoon ISF		Evening ISF	
	Correlation coefficient	*p*-value	Correlation coefficient	*p*-value	Correlation coefficient	*p*-value
Age (years)	–0.82	< 0.001	–0.78	< 0.001	–0.81	< 0.001
Sex	0.03	0.81	–0.06	0.59	0.06	0.55
Tanner stage	–0.87	< 0.001	–0.83	< 0.001	–0.85	< 0.001
BMI (kg/m^2^)	–0.79	< 0.001	–0.79	< 0.001	–0.078	< 0.001
Duration of diabetes (years)	–0.26	0.01	–0.28	0.005	–0.26	0.012
TDD (units/day)	–0.96	< 0.001	–0.95	< 0.001	–0.96	< 0.001
ICR (grams/unit) for the same time of the day	0.98	< 0.001	0.93	< 0.001	0.94	< 0.001
HbA1c (%)	–0.06	0.55	–0.08	0.41	–0.08	0.41

*BMI, body mass index; HbA1c, glycosylated hemoglobin A1c; ICR, insulin-to-carbohydrate ratio; ISF, insulin sensitivity factor; TDD, total daily insulin dose.*

**TABLE 6:** Multiple regression analyses for factors associated with insulin sensitivity factors at different times of the day.

Variables	Morning ISF		Afternoon ISF		Evening ISF	
	Estimate (95% CI)	*p*-value	Estimate (95% CI)	*p*-value	Estimate (95% CI)	*p*-value
Age (years)	−0.18 (−0.60: 0.24)	0.41	−0.42 (−0.93: 0.08)	0.10	−0.41 (−0.94: 0.12)	0.13
BMI (kg/m^2^)	−0.34 (−0.68: −0.01)	0.05	−0.27 (−0.76: 0.22)	0.28	−0.29 (−0.75: 0.16)	0.21
Duration of diabetes (years)	−0.25 (−0.56: 0.06)	0.11	−0.30 (−0.73: 0.13)	0.17	−0.38 (−0.79: 0.03)	0.07
ICR (grams/unit) for the same time of the day	4.56 (4.14: 4.98)	<0.001	4.38 (3.90: 4.86)	<0.001	4.45 (3.99: 4.92)	<0.001

*BMI, body mass index; CI, confidence interval; ICR, insulin-to-carbohydrate ratio; ISF, insulin sensitivity factor.*

**FIGURE 1:**
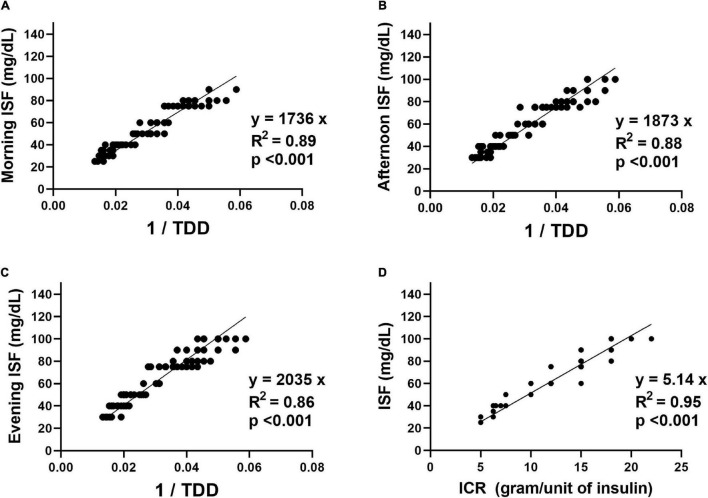
The relationships between the insulin sensitivity factor (ISF) in mg/dL for the study participants and their insulin-to-carbohydrate ratio (ICR) in grams/unit of insulin and total daily insulin dose (TDD) in units/day. **(A)** The relationship between the morning ISF and the reciprocal of the TDD. **(B)** The relationship between the afternoon ISF and the reciprocal of the TDD. **(C)** The relationship between the evening ISF and the reciprocal of the TDD. **(D)** The relationship between the ISF for different times of the day and the ICR for the same time of the day.

The relationships between ISF for the study participants and their ICR and TDD are shown in Figure 1. Linear regression analyses were used to derive formulas for ISF estimation. The formulas derived from the linear relationships between ISF (in mg/dL) for different times of the day and the reciprocal of TDD (unit/day) with the y-intercept set at zero revealed that the morning ISF could be estimated as 1736/TDD (coefficient = 1736, 95% confidence interval (CI): 1699–1774, *R*^2^ = 0.89, *p* < 0.001), the afternoon ISF could be estimated as 1873/TDD (coefficient = 1873, 95% CI: 1829–1916, *R*^2^ = 0.88, *p* < 0.001) and the evening ISF could be estimated as 2035/TDD (coefficient = 2035, 95% CI: 1986–2083, *R*^2^ = 0.86, *p* < 0.001). The formula derived from the linear relationship between ISF and ICR for different times of the day with the y-intercept set at zero revealed that ISF (in mg/dL) for any time of the day could be estimated as 5.14 × ICR for the same time of the day (coefficient = 5.14, 95% CI: 5.10–5.19, *R*^2^ = 0.95, *p* < 0.001).

## Discussion

The current study demonstrated that ISF and ICR in children and adolescents with T1DM using ultra-long-acting basal insulin showed diurnal variation with higher bolus insulin requirements in the morning than in the afternoon and the evening. A similar pattern of diurnal variation of ISF and ICR among prepubertal children with T1DM using insulin pumps was reported by Hanas et al., who found that higher bolus insulin requirements both as pre-meal doses and as correction doses were needed for the morning than for the rest of the day (9).

The increased insulin requirement in the morning might be related to the peak of growth hormone secretion. Growth hormone has an anti-insulin effect through increasing hepatic glucose synthesis and decreasing glucose uptake by peripheral tissue (30). Growth hormone has pulsatile secretion throughout the day but about 50% of its secretion occurs at night (42). The dawn phenomenon which is characterized by increased blood glucose levels in the early morning hours is preceded by the peak of growth hormone secretion which occurs around 3 AM (43). The increased insulin resistance induced by growth hormone secretion usually begins around 4 AM reaching its peak at 8–10 AM (44).

The study demonstrated that the 1800 rule was appropriate for calculating the correction insulin dose requirements in the morning. However, the actual correction dose requirements for the afternoon and the evening were significantly lower than those estimated by the 1800 rule. Using linear regression analyses between ISF at different times of the day and the TDD for the participants, the study proposed that ISF (in mg/dL) could be estimated as 1736/TDD for the morning, 1873/TDD for the afternoon and 2035/TDD for the evening. In line with these findings, Hanas et al., reported that the actual correction doses required by prepubertal children using insulin pumps were lower than those estimated by the 1800 rule (9). Moreover, Lau et al., conducted a retrospective study on 292 children and adolescents with T1DM using insulin pumps and they found that the real-life ISF detected in their study participants were about 124% and 111% of those calculated by the 1800 rule for prepubertal children and adolescents, respectively (7).

Despite the results of these studies, the 1800 rule is widely used for ISF estimation among children and adolescents with T1DM using insulin pumps (1). Moreover, new insulin pumps that provide automated insulin delivery utilize the 1800 rule in the algorithms for automated correction doses calculation (45). However, these insulin pumps deliver the correction dose as micro-boluses every 5 min guided by the real-time continuous glucose monitoring (CGM) transmitted glucose values (45). Furthermore, the insulin pumps take into account the duration of active insulin and adjust insulin delivery according to the amount of active insulin on board (46). These measures protect against hypoglycemia if the calculated correction dose was slightly higher than the required dose. On the other hand, patients using the MDI regimen usually get the correction dose as a single shot. Moreover, calculating the amount of active insulin remaining from the previous bolus dose with each bolus injection might be difficult for patients using the MDI regimen. This makes those patients more likely to develop severe hypoglycemia if they had slightly higher correction doses than they need.

The current study found that the diurnal variation of ISF was found in participants using insulin glargine 300 Units/mL as well as those using insulin degludec 100 Units/mL. However, the study did not find significant differences in the TDD, the daily basal insulin dose, ICR or ISF at different times of the day between participants using insulin glargine 300 Units/mL and those using insulin degludec 100 Units/mL. Some previous studies reported higher daily insulin doses in patients shifted to insulin glargine 300 U/mL (47, 48) and lower daily insulin doses in patients shifted to insulin degludec 100 U/ml (49, 50) compared to their doses on insulin glargine 100 U/mL. However, the findings of the current study were in line with the results of some recent studies comparing the TDD between patients using insulin glargine 300 U/mL and those using insulin degludec 100 U/mL. These studies reported that there was no significant difference in the TDD between patients using either type of ultra-long-acting basal insulin analogs (51, 52).

The current study demonstrated that the diurnal variation pattern of ISF with higher correction doses requirements in the morning compared to the afternoon and the evening was found in participants with different pubertal stages. Moreover, the study showed that ISF for different times of the day were significantly lower with higher correction doses requirements in late-pubertal and postpubertal participants compared to prepubertal and early to mid-pubertal participants. Similarly, Cemeroglu et al., demonstrated the effect of age and pubertal status on ISF in a retrospective study conducted on 154 well-controlled T1DM pump users aged between 3 and 21 years. They found that ISF decreased with age resulting in more insulin requirements for correction doses in adolescents and adults compared to younger children (6). Furthermore, Andersen et al., conducted a retrospective study on 124 children and adolescents with well-controlled T1DM using insulin pumps (8). They found that ISF in adolescents was lower than that for younger children. This decreased insulin sensitivity in adolescents might be attributed to the effect of hormonal changes during puberty (16).

The present study demonstrated that the ISF at different times of the day were significantly correlated with the age, Tanner stage, BMI, duration of diabetes, TDD, and ICR. Multivariate analysis of factors significantly correlated with ISF revealed that ICR was the most significant factor associated with ISF. Similarly, Andersen et al., studied factors associated with ISF in children and adolescents using insulin pumps. They found that ISF was significantly associated with age, TDD, the amount of carbohydrates in the diet and the duration of pump therapy (8).

Using the linear relationship between ISF and ICR among the study participants, the current study proposed that ISF (in mg/dL) for any time of the day could be estimated as 5.14 × ICR for the same time of the day. The linear relationship between ICR and ISF was also demonstrated by Alemzadeh et al., in a 1-year prospective study on 14 young children with T1DM using insulin pumps. They reported strong relationships between ICR, ISF, TDD, and basal doses and that any adjustment for one factor required modifications for the others (5). In addition, King and Armstrong in a 2-week prospective study on T1DM adults using insulin pumps found a strong linear relationship between ISF and ICR and proposed that ISF (in mg/dL) could be estimated as 4.44 × ICR (53). However, the difference between the formula proposed by King and Armstrong and the one proposed by the current study might be attributed to differences in BMI and changes in insulin sensitivity with age.

The strength of the present study is that it prospectively assessed the real-life ISF in children and adolescents using the MDI regimen with ultra-long-acting basal insulin analogs. Studies on ISF and ICR among children and adolescents with T1DM using the MDI regimen are still scarce although those patients represent the majority of pediatric patients with T1DM especially in countries where the resources of the healthcare systems cannot afford the high costs of insulin pump therapy (54).

However, the study had some limitations. First, it was a single-center study. Larger multicenter studies are required to confirm the findings of this study. Second, blood glucose levels in the study participants were measured using finger-stick glucometers and not by CGM devices. Therefore, variations in blood glucose levels throughout the day were not always available.

## Conclusion

The diurnal variation of ISF has to be considered when calculating correction insulin doses for children and adolescents with T1DM using the MDI regimen with ultra-long-acting basal insulin analogs. The diurnal variation pattern of higher bolus insulin requirement in the morning compared to the rest of the day was found in children and adolescents with different pubertal stages. The 1800 rule was appropriate for calculating correction doses in the morning but not for the afternoon or the evening. Both the TDD and the ICR could be used for ISF estimation.

## Data Availability Statement

The original contributions presented in the study are included in the article/supplementary material, further inquiries can be directed to the corresponding author.

## Ethics Statement

The studies involving human participants were reviewed and approved by the Research Ethics Committee at Sohag Faculty of Medicine, Sohag University. Written informed consent to participate in this study was provided by the participants’ legal guardian/next of kin.

## Author Contributions

AH designed the study, collected and analyzed the data, and wrote the manuscript.

## Conflict of Interest

The author declares that the research was conducted in the absence of any commercial or financial relationships that could be construed as a potential conflict of interest.

## Publisher’s Note

All claims expressed in this article are solely those of the authors and do not necessarily represent those of their affiliated organizations, or those of the publisher, the editors and the reviewers. Any product that may be evaluated in this article, or claim that may be made by its manufacturer, is not guaranteed or endorsed by the publisher.
